# Possible associations of personality traits representing harm avoidance and self-directedness with medication adherence in Japanese patients with type 2 diabetes

**DOI:** 10.1186/s40780-018-0112-4

**Published:** 2018-07-03

**Authors:** Yoshiko Tominaga, Tohru Aomori, Tomohisa Hayakawa, Nobuhiko Kijima, Donald E. Morisky, Kunihiko Takahashi, Mayumi Mochizuki

**Affiliations:** 1 Division of Hospital Pharmacy Science, Graduate School of Pharmaceutical Sciences, Keio University, 1-5-30 Shibakoen, Minato-ku, Tokyo, 105-8512 Japan; 2 Division of Hospital Pharmacy Science, Faculty of Pharmacy, Keio University, 1-5-30 Shibakoen, Minato-ku, Tokyo, 105-8512 Japan; 3 Psychological Laboratory, Keio University, 4-1-1 Hiyoshi, Kohoku-ku, Yokohama, 223-8521 Japan; 4 Department of Community Health Sciences, UCLA Fielding School of Public Health, 650 Charles E. Young Drive South, Los Angeles, CA 90095-1772 USA; 5 Department of Clinical Epidemiology and Biostatistics, Graduate School of Medicine, Nagoya University, 65 Tsurumai-cho, Showa-ku, Nagoya, 466-8550 Japan

**Keywords:** Medication adherence - personality trait - temperament and character inventory -harm avoidance - self-directedness - Morisky medication adherence scale

## Abstract

**Background:**

Insufficient medication adherence in diabetes patients, of which numbers continue to increase globally, remains a critical issue. Medication adherence is multifactorial and determined by interactions among factors including socioeconomic status, health care team and system, condition, therapy, and patient-specific factors. On the other hand, personality traits have been studied in adherence other than to medication. Using the instruments of Temperament and Character Inventory (TCI), Harm Avoidance (TCI-HA) and Self-directedness (TCI-SD) showed distinguishing associations with adherence of health-related programs. However, few studies have been performed to elucidate psychometric properties related to medication adherence. We investigated how TCI-HA and TCI-SD of patients with diabetes are related to medication adherence.

**Method:**

A cross-sectional survey was conducted among type 2 diabetes patients recruited at medical institutions or via an online research company. Medication adherence was measured using the 8-item Morisky Medication Adherence Scale (MMAS-8). Personality traits were assessed using the established scales of TCI-HA and TCI-SD. Univariate and multivariate regression analyses of the MMAS-8 scores were performed in addition to assessing demographic and disease characteristics and TCI-HA and TCI-SD.

**Results:**

A total of 358 responses were analyzed. Multivariate regression analysis of MMAS-8 scores revealed that higher TCI-SD was related to better adherence and experiencing drug-related side effects was related to poor adherence. Aging was significantly associated with better medication adherence in univariate regression analysis but became insignificant in multivariate regression.

**Conclusions:**

In diabetes patients, the anxiety reflected in TCI-HA tends to lower and the self-control reflected in TCI-SD tends to promote medication adherence. TCI-SD has a greater effect than TCI-HA.

**Electronic supplementary material:**

The online version of this article (10.1186/s40780-018-0112-4) contains supplementary material, which is available to authorized users.

## Background

The population of diabetic patients worldwide reached 415 million in 2015, an increase of 28 million from the previous year, and the prevalence rate was 8.8% among those between the ages of 20 and 79 years [1]. The diabetic population in Japan was 3.16 million in 2014, among the highest in the periodic national surveys performed every three years [2]. According to a systematic review of medication adherence among diabetic patients [3], the adherence rate varied from 38.5 to 93.1% and only six of 27 studies (22.2%) reported 80% or higher rates of adherence in their target populations. Diabetes patients not only forget taking medications but also deliberately omit for some reasons: e.g. the disease is perceived as stable [4]. It is globally recognized that poor medication adherence not only affects clinical outcomes adversely but is also a critical issue from a health economic perspective [5].

Medication adherence is in general multifactorial and determined by interactions among factors related to socioeconomic status, the healthcare team and system, condition severity, type of therapy, and individual patient characteristics [5]. Various studies evaluated factors associated with medication adherence in diabetes patients, including: gender [6, 7]; age [6–10]; medication complexity [11]; depression [8, 11, 12]; and healthcare costs [11, 13]. Self-efficacy, defined as the belief that one can successfully perform the behavior required to produce a given outcome [14], was also demonstrated to be related to medication adherence [15]. Qualitative research in elderly Japanese patients identified “balancing positive and negative thoughts” as a central concept for medication adherence [16]. Those findings imply that underlying factors characterizing specific individual personalities might be pertinent to medication behavior and attitude. However, few studies have been performed to elucidate psychometric properties in relation to medication adherence using validated tools.

In psychology field, personality traits have been studied in non-patients as well as patients with mental illness or some others. The personality traits are measured by specific instruments, one of which is Temperament and Character Inventory (TCI) developed by Cloninger [17], consists 7 dimensions. Using TCI, some studies examined personality traits specific to adherence other than to medication. The attrition rate of obese patients participating in a weight loss program was associated with Harm Avoidance (TCI-HA), Reward Dependence, and Novelty Seeking of the seven [18]. The abandonment and relapse rates of alcoholic patients undergoing detoxification treatment done was associated with Self-Directedness (TCI-SD) and Cooperativeness of the seven [19]. Use of continuous positive air-pressure devices in Japanese patients with sleep apnea syndrome was associated with TCI-HA and TCI-SD [20]. TCI-HA reflects anticipatory worry and pessimism, fear of uncertainty, shyness, and fatigability and TCI-SD reflects feelings of responsibility, purposefulness, resourcefulness, self-acceptance, and congruency, TCI-HA and TCI-SD were also observed to be associated with depression [21, 22], which is a known factor of affecting medication adherence [8, 11, 12]. Therefore, we hypothesized TCI-HA and TCI-SD be also relevant to adherence of medication. If it is confirmed, we could utilize such personality traits to predict poor adherence of medication in implementing effective intervention programs for better adherence.

In this study, we attempted to investigate how the selected personality traits, TCI-HA and TCI-SD, of patients with diabetes are related to medication adherence.

## Participants and methods

### Setting

The study participants were patients with type 2 diabetes receiving pharmacotherapy. They were recruited from among those regularly visiting hospitals or community pharmacies (direct channel) or those voluntarily registering with a Japanese online research company (indirect channel), as utilized for surveys in healthcare fields [23–25], between December 2016 and April 2017.

In the direct channel, medical professionals at hospitals or community pharmacies who were able to confirm the diagnoses of patients with type 2 diabetes were in charge of recruitment. They obtained informed consent from participants and provided them with the Form A questionnaire, which consists of items covering personality traits, medication adherence, height, body weight, duration of diabetes from diagnosis (diabetes duration), experience of hypoglycemia or drug-related side effects, and family history of diabetes. The participants filled out the questionnaires, placed them in the designated envelopes, and returned them to the medical professionals or sent them to the researchers via postal mail in order to minimize potential social desirability bias. The medical professionals filled out the Form B questionnaire on medication content and glycosylated hemoglobin (HbA1c) levels.

In the indirect channel, the company, which operates a database with approximately 300,000 patient registrants including more than 7000 with type 2 diabetes, sent invitations to participate to random target patients. The patients indicated consent to participate in the survey and responded online to the questions, which were comparable to the items in the From A and Form B questionnaires in the direct channel. Collection of the responses was closed when it was calculated that around evaluable 150 samples would have been received. The responses were checked by the company for technical errors or inappropriate input from an administrative standpoint. The dual-channel recruitment method was employed because of the assumed age distribution in each, i.e., patients in the direct channel tended to be older and those in the indirect one to be younger, and thus the differences were expected to be complementary.

The Ethics and Research Board of the Faculty of Pharmacy, Keio University, approved this study protocol (approval nos. 161,215–1 and 170,120–1).

### Assessments

Medication adherence was assessed using the validated Japanese version of 8-item Morisky Medication Adherence Scale (MMAS-8) [26–29], since it is widely used and confirmed to be one of the most reliable self-administered questionnaires available [30]. Among the 8 questions (Table 1), 7 are answered with “yes” or “no” and the 8th is answered with a 5-item Likert scale. Responses were coded by a designated rule and calculated for total scores between 0 and 8, with 8 being the most adherent.

**Table 1:** Question Items of Medication Adherence (MMAS-8)^a)^

1. Do you sometimes forget to take your diabetic medication(s)?	
2. People sometimes miss taking their medications for reasons other than forgetting. Thinking over the past two weeks, were there any days when you did not take your diabetic medication (s)?	
3. Have you ever cut back or stopped taking your medication(s) without telling your doctor, because you felt worse when you took it?	
4. When you travel or leave home, do you sometimes forget to bring along your diabetic medication (s)?	
5. Did you take your diabetic medication (s) yesterday?	
6. When you feel like your blood glucose is under control, do you sometimes stop taking your medication (s)?	
7. Taking medication (s) every day is a real inconvenience for some people. Do you ever feel hassled about sticking to your diabetic treatment plan?	
8. How often do you have difficulty remembering to take all your medication (s)?	

^a)^MMAS-8: 8-item version of Morisky Medication Adherence Scale. The MMAS (8-item) content, names and trademarks are protected by US copyright and trademark laws. Permission for use of the scale and its coding is required. A license agreement is available from: Donald E. Morisky, ScD, ScM, MSPH, 14725 NE 20th St Bellevue, WA 98007, USA; dmorisky@gmail.com

Personality traits were assessed using the validated Japanese version of TCI. In this study, receiving prior permission from the developer, we utilized two dimensions TCI-HA and TCI-SD. The total 40 questions, 20 of each dimension, were answered on a 5-point scale. Responses were coded by a designated rule and calculated for total scores in the range of 5–100 of each.

Glycemic control was represented by averaging at least three testing points of HbA1c obtained during the previous 12 months. Demographic and disease characteristics collected as background variables were age, gender, body mass index (BMI), duration of diabetes from diagnosis (diabetes duration), insulin use, number of diabetic medications, experience of hypoglycemia or drug-related side effects, and family history of diabetes. Questionnaires filled out by the participants in the direct channel were sent directly to the researchers without being seen by the medical professionals to minimize the potential social desirability bias.

### Statistical analysis

The basic statistical model assumed that demographics, disease characteristics, and TCI-HA/TCI-SD might affect MMAS-8 scores. We conducted both univariate and multivariate regression analyses for MMAS-8 as the continuous dependent variable. Independent variables were 11 in total: gender, age, BMI, diabetes duration, number of diabetic medications, insulin use (yes/no), experience of hypoglycemia (yes/no), experience of drug-related side effects (yes/no), family history of diabetes (yes/no), TCI-HA, TCI-SD, and recruiting method (direct/indirect). For multivariate adjusted model for MMAS-8 score, all the independent variables were unconditionally entered in the linear regression model, and a bidirectional stepwise algorithm was used on a threshold condition of *p* value at 0.1. Separately, multivariate full model of regression analysis for MMAS-8 was employed.

Dependent variables in regression analyses were indicated with standardized coefficients (beta), 95% confidence intervals (CIs), and *p* values. All *p* values of less than 0.05 (2-sided test) were regarded as representing statistically significant differences. The JMP 13 (SAS Institute Inc., Cary, NC, USA) was used for all data analyses.

## Results

### Study population

In the directed channel, 355 of Form A questionnaire were distributed to patients and 221, together with Form B corresponding to these patients, were collected. In the indirect channel, 149 responses were collected. A total of 370 patients responded to the questionnaires, of which 358 were included in the final analysis. The 12 patients were excluded since they were incomplete on either of the essential items: MMAS-8 score (4), HbA1c level (5), TCI-HA/TCI-SD (3). The demographic and disease characteristics of the participants analyzed, in addition to MMAS-8 scores, as the total and breakdown by the recruiting channels, are shown in Table 2. Two hundred and eighteen (61%) were men, and the mean (± SD) age was 60.3 ± 11.7 years. Those who enrolled via the indirect channel included more male and were younger and less often to use insulin and to have family members with diabetes than those via the direct channel.

**Table 2:** Participants’ background characteristics

	Total (*n* = 358)	Direct Channel (*n* = 212)	Indirect Channel (*n* = 146)
Male, *n* (%)	218 (61)	116 (55)	102 (70)
Age (years, mean ± SD)	60.3 ± 11.7	64.3 ± 11.7	54.6 ± 9.5
BMI (%, mean ± SD)	25.5 ± 5.2	24.7 ± 4.6	26.8 ± 5.8
Diabetes duration (years, mean ± SD)	12.2 ± 8.9	12.7 ± 9.6	11.4 ± 7.8
Number of medications (mean ± SD)	2.1 ± 1.1	2.3 ± 1.1	1.9 ± 1.2
Insulin use, *n* (%)	78 (22)	54 (26)	24 (17)
Hypoglycemia (within past year), *n* (%)	85 (24)	55 (26)	30 (21)
Drug-related side effects, *n* (%)	84 (23)	50 (24)	34 (23)
Diabetes patients in family, *n* (%)	177 (50)	115 (55)	62 (42)
TCI-HA score (mean ± SD)^a)^	59.6 ± 13.4	58.1 ± 14.0	61.7 ± 12.4
TCI-SD score (mean ± SD)^b)^	56.6 ± 10.0	59.4 ± 9.6	53.0 ± 9.6
MMAS-8 score (mean ± SD)^c)^	6.5 ± 1.6	6.6 ± 1.5	6.4 ± 1.7
HbA1c level, % (mean ± SD)	7.2 ± 1.0	7.3 ± 1.0	7.2 ± 1.1

^a)^TCI-HA: Harm Avoidance in the Temperament and Character Inventory

^b)^TCI-SD: Self-directedness in the Temperament and Character Inventory

^c)^MMAS-8: 8-item version of Morisky Medication Adherence Scale

### Factors associated with medication adherence

Univariate regression analysis for MMAS-8 score (Additional file 1: Table S1) revealed that age (beta 0.13, CI 0.00 to 0.03, *p* = 0.017) and TCI-SD (beta 0.15, 95% CI 0.01 to 0.04, *p* = 0.006) were positively correlated, and the number of medications (beta − 0.11, 95% CI –0.31 to − 0.02, *p* = 0.028), experience of drug-related side effects (beta − 0.18, 95% CI –0.52 to − 0.14, *p* = 0.001), and TCI-HA (beta − 0.11, 95% CI –0.03 to − 0.00, *p* = 0.033) were negatively correlated with MMAS-8 scores. The result of adjusted model of multivariate regression analysis for MMAS-8 score was presented in Table 3, TCI-SD (beta 0.12, 95% CI 0.00 to 0.04, *p* = 0.037) was positively, and experience of drug-related side effects (beta − 0.14, 95% CI –0.46 to − 0.06, *p* = 0.011) was negatively correlated with MMAS-8 scores. The difference in recruiting channels was not significantly associated with MMAS-8 in either univariate or multivariate regression analyses. The full model of multivariate regression analysis for MMAS-8 score was shown in Additional file 1: Table S1.

**Table 3:** Factors Associated with Medication Adherence (MMAS-8)^a)^

Independent variables	Multivariate (Adjusted model)^b)^		
	Beta^c)^	95% CI	*p* value
Age (years)	0.09	(−0.00, 0.03)	0.137
Number of medications	−0.11	(−0.29, 0.00)	0.050
Drug-related side effects: Yes	−0.14	(−0.46, − 0.06)	0.011
Diabetes patients in family: Yes	−0.08	(− 0.29, 0.04)	0.151
TCI-SD score^d)^	0.12	(0.00, 0.04)	0.037

^a)^MMAS-8: 8-item version of Morisky Medication Adherence Scale

^b)^Multivariate regression analysis: all the independent variables were unconditionally entered in both of the linear models, and a bidirectional stepwise algorithm was used on a threshold condition of *p* value at 0.1

^c)^Standardized coefficient of regression model

^d)^TCI-SD: Self-directedness in the Temperament and Character Inventory

## Discussion

This was the first attempt to examine diabetic patients’ medication behavior from the personality standpoint. Based on the present results showing associations with some TCI dimensions, we hypothesized that TCI-HA decreases and TCI-SD promotes medication adherence and demonstrated that preliminarily in this study. Various methodological options exist for measuring medication adherence in general: electronic monitoring of sensor-embedded containers; manually counting the tablets/pills remaining in containers by medical professionals; and biological quantification of serum-level concentrations. However, none is technically or economically feasible in the real-world setting [30]. Furthermore, patients consciously or unconsciously may not give accurate accounts of their medication status, while physicians do not necessarily note it [31]. Among the Japanese diabetes patients in the community pharmacy setting, their self-reporting of medication adherence was consistently overrated when compared with the results found when pharmacists counted the remaining tablets or pills [32]. Diabetes, unlike acute curable diseases, requires long-term patient self-management of medication, diet, exercise, and possibly self-injection together with blood glucose monitoring as part of daily life. It is recognized that the general thinking and behavior of each patient influence medication adherence. In terms of the magnitude of the influence of these two factors, TCI-SD may have a somewhat greater effect than TCI-HA since the former remained valid in multivariate regression analysis of MMAS-8 scores. Based on the finding that those who score relatively high in TCI-HA and/or low in TCI-SD are potentially at risk of noncompliance with medication protocols, medical professionals should take that into consideration when they note signs of that personality type in patients’ daily behavior and attitudes.

In multivariate regression analysis involving MMAS-8 scores, taking more medications showed a tendency to be negatively associated with better medication adherence, as found in an earlier study [11]. Those who receive more prescriptions may have a greater burden in terms of increased complexity in taking their medications. This can cause lower adherence and, as shown in this study, eventually poor glycemic control. Experiencing drug-related side effects was also negatively correlated with medication adherence, consistent with the results of a previous study [12]. This is possibly because patients who experience side effects may fear that they will recur and become reluctant to continue the causative medication. Such anxiety is an attribute of TCI-HA and is thus consistent with the observation in this study that TCI-HA is negatively associated with medication adherence. The factor of age was significantly correlated with the MMAS-8 score in univariate regression analysis but became insignificant in multivariate regression, partly due to multiple co-linearity with the remaining variables. Age was significantly correlated with BMI (*r* = − 0.38, *p* < 0.001), diabetes duration (*r* = 0.33, *p* < 0.001), TCI-HA (*r* = − 0.27, *p* < 0.001), and TCI-SD (*r* = 0.36, *p* < 0.001).

Older age is often noted to have a distinct relationship with medication adherence [6–10]. In a systematic review [3], 10 of 17 studies revealed that increasing age was positively associated with adherence but there was little discussion of the reason for that. The present study also showed that older age was significantly associated with better adherence in univariate regression analysis. The basis of an individual’s personality is formed in childhood and adolescence and gradually develops until around the 30s. There is debate on whether development ceases in the 30s or continues even in maturity. TCI-SD was observed to correlate with aging even after the 30s [33, 34]. A meta-analysis found that personality traits using some scales showed a clear pattern of normative change throughout the life course, although such changes in old age were not as great as those in adolescence and young adulthood [35]. In the present study, advancing age was related to higher TCI-SD and lower TCI-HA in the age range of 30 to 70 years, as noted above. Although it can be speculated that the elderly are better adherents due to the personality traits reflected by TCI-SD and TCI-HA, further research is needed to reach a firm conclusion.

By utilizing the dual-channel method of recruiting participants, this study included sufficient representation of both younger and older patients with diabetes. As assumed in advance, the mean age of participants recruited via the direct and indirect channels was 64.2 ± 11.6 and 54.5 ± 9.4 years, respectively. However, the recruiting channel factor did not show a significant association with medication adherence in either univariate or multivariate regression analyses (Additional file 1: Table S1), and thus it was regarded as appropriate to consolidate the results in these two populations.

### Limitations

There were two limitations in this study. First, the questionnaire responses on HbA1c levels and medication regimens of participants recruited via the research company were completed by themselves. Although they were requested to respond honestly to the questionnaire based on their own documented health records, it was not possible to ensure the validity of the responses by an independent source. Second, we limited to the two traits of TCI-HA and TCI-SD from the total of seven in the TCI based on our scientific rationale and did not investigate the other five traits primarily for the purpose of minimizing participants’ burden of responding to many questions. Consequently, the effects of the other five were not explored.

## Conclusion

Personality traits relevant to anxiety tend to lower and traits relevant to self-control tend to increase adherence to medication regimens in patients with diabetes. The influence of the latter is greater than that of the former. Medication behaviors and attitudes and glycemic control levels are affected by current medication and patients’ experience with pharmacotherapy. Aging is likely to modify medication adherence in combination with the effects of other modifiers.

## Additional file

Additional file 1: **Table S1.** Factors Associated with Medication Adherence (MMAS-8)^a)^. (DOCX 18 kb)

## Abbreviations

BMI: body mass index; beta: standardized coefficients
CI: confidence interval
HA: Harm Avoidance
HbA1c: glycosylated hemoglobin
MMAS-8: 8-item Morisky Medication Adherence Scale
SD: Self-directedness
TCI: Temperament and Character Inventory
