# Supplementary material for: Possible associations of personality traits representing harm avoidance and self-directedness with medication adherence in Japanese patients with type 2 diabetes
Source: J Pharm Health Care Sci. 2018 Jul 3;4:16. doi: 10.1186/s40780-018-0112-4 (PMC6029079; doi:10.1186/s40780-018-0112-4)
Supplement: Supplementary file 1 — Table S1. Factors Associated with Medication Adherence (MMAS-8)a). (DOCX 18 kb) [file 40780_2018_112_MOESM1_ESM.docx]

**Table S1 Factors Associated with Medication Adherence (MMAS-8)**^a)^

| Independent variables | Univariate | | | Multivariate (full model) | | |
| --- | --- | --- | --- | --- | --- | --- |
|  | Beta^b)^ | 95% CI | *p* value | Beta^b)^ | 95% CI | *p* value |
| Gender: Male | –0.03 | (–0.22, 0.12) | 0.579 | –0.08 | (–0.31, 0.06) | 0.177 |
| Age (years) | 0.13 | (0.00, 0.03) | 0.017 | 0.10 | (–0.01, 0.03) | 0.154 |
| BMI (%) | –0.03 | (–0.04, 0.02) | 0.539 | 0.03 | (–0.03, 0.04) | 0.603 |
| Diabetes duration (years) | 0.00 | (–0.02, 0.02) | 0.174 | –0.01 | (–0.02, 002) | 0.816 |
| Number of medications | –0.11 | (–0.31, –0.02) | 0.028 | –0.09 | (–0.28, 0.03) | 0.117 |
| Insulin use: Yes | 0.09 | (–0.02, 0.37) | 0.083 | 0.06 | (–0.12, 0.33) | 0.360 |
| Hypoglycemia (within past year): Yes | 0.06 | (–0.09, 0.30) | 0.296 | –0.02 | (–0.25, 0.19) | 0.775 |
| Drug–related side effects: Yes | –0.18 | (–0.52, –0.14) | 0.001 | –0.14 | (–0.47, –0.05) | 0.013 |
| Diabetes patients in family: Yes | 0.10 | (–0.01, 0.31) | 0.074 | –0.10 | (–0.34, 0.02) | 0.080 |
| TCI–HA score^c)^ | –0.11 | (–0.03, –0.00) | 0.033 | –0.04 | (–0.02, 0.01) | 0.607 |
| TCI–SD score^d)^ | 0.15 | (0.01, 0.04) | 0.006 | 0.08 | (–0.01, 0.01) | 0.287 |
| Recruiting channel: Indirect | –0.06 | (–0.26, 0.08) | 0.280 | –0.05 | (–0.28, 0.12) | 0.438 |

^a)^MMAS-8: 8-item version of Morisky Medication Adherence Scale.

^b)^Standardized coefficient of regression model.

^c)^TCI-HA: Harm Avoidance in the Temperament and Character Inventory.

^d)^TCI-SD: Self-directedness in the Temperament and Character Inventory.
